# Patient Characteristics and Outcomes of Outpatient Parenteral Antimicrobial Therapy: A Retrospective Study

**DOI:** 10.1155/2016/8435257

**Published:** 2016-02-22

**Authors:** Marie Yan, Marion Elligsen, Andrew E. Simor, Nick Daneman

**Affiliations:** ^1^Faculty of Medicine, University of Toronto, 1 King's College Circle, Toronto, ON, Canada M5S 1A8; ^2^Division of Infectious Diseases, Sunnybrook Health Sciences Centre, 2075 Bayview Avenue, Toronto, ON, Canada M4N 3M5; ^3^Institute for Clinical Evaluative Sciences, 2075 Bayview Avenue, Toronto, ON, Canada M4N 3M5

## Abstract

Outpatient parenteral antimicrobial therapy (OPAT) is a safe and effective alternative to hospitalization for many patients with infectious diseases. The objective of this study was to describe the OPAT experience at a Canadian tertiary academic centre in the absence of a formal OPAT program. This was achieved through a retrospective chart review of OPAT patients discharged from Sunnybrook Health Sciences Centre within a one-year period. Between June 2012 and May 2013, 104 patients (median age 63 years) were discharged home with parenteral antimicrobials. The most commonly treated syndromes included surgical site infections (33%), osteoarticular infections (28%), and bacteremia (21%). The most frequently prescribed antimicrobials were ceftriaxone (21%) and cefazolin (20%). Only 56% of the patients received follow-up care from an infectious diseases specialist. In the 60 days following discharge, 43% of the patients returned to the emergency department, while 26% required readmission. Forty-eight percent of the return visits were due to infection relapse or treatment failure, and 23% could be attributed to OPAT-related complications. These results suggest that many OPAT patients have unplanned health care encounters because of issues related to their infection or treatment, and the creation of a formal OPAT clinic may help improve outcomes.

## 1. Introduction

Outpatient parenteral antimicrobial therapy (OPAT) is a treatment option that enables patients to receive parenteral antimicrobials at home. Since it was introduced nearly 40 years ago, numerous studies have established OPAT as a safe and effective alternative to continued hospitalization, and many institutions have implemented formal OPAT clinics and services [1–4]. OPAT offers a number of advantages to both the patient and the health care provider. Because patients are able to recuperate at home, they experience accelerated psychological and physical recovery and report improved patient satisfaction. Early discharge also reduces the risk of nosocomial infections [4, 5]. From the institutional perspective, OPAT is highly cost-effective and facilitates the efficient use of health care resources by increasing bed availability [2, 5].

Guidelines for OPAT have been published for both adult and paediatric populations and include specific recommendations for patient and drug selection as well as follow-up. Diligent patient monitoring is suggested because of the risk of treatment failure, antimicrobial complications, or other adverse events [4, 6, 7]. These outcomes can result in unplanned readmission or emergency department visits during OPAT [8–10]. Some factors that have been found to be associated with increased readmission include age, type of antimicrobial resistant, prior history of antibiotic-resistant organisms, and poor access to laboratory monitoring test results [11, 12].

There is limited information about the prevalence of existing OPAT clinics in Canada; however, formal and informal OPAT programs have been described for select hospitals in Vancouver (British Columbia), Calgary (Alberta), and Manitoba [2, 13, 14]. To our knowledge, specialized OPAT clinics remain relatively uncommon in Ontario, where OPAT is usually prescribed and managed at the discretion of the individual physician. As a result, very little is known about the frequency of OPAT use or the characteristics and outcomes of these patients. Therefore, the objective of the present study was to describe the OPAT population at a Canadian tertiary hospital in the absence of a formal OPAT program.

## 2. Methods

This study was a single-centre retrospective cohort study conducted at Sunnybrook Health Sciences Centre (SHSC), an academic tertiary facility located in Toronto, Ontario. The selected patient population consisted of all SHSC patients (aged 18 and above) who were discharged home with parenteral antimicrobial treatment between June 1, 2012 and May 31, 2013. Eligible patients were identified by merging two databases: (A) the list of patients who were referred for home care services through Ontario's Community Care Access Centres (CCACs) and (B) the pharmacy list of patients with parenteral antimicrobials “active” on the day of discharge. During chart review, we excluded patients whose antimicrobials had not been continued in the outpatient setting. We also excluded patients who were transferred to other acute care, long-term care, or rehabilitation facilities after discharge. The patients in this study were not managed through a dedicated OPAT clinic, and parenteral therapy was administered by home care nurses either at home or in CCAC clinics.

Patient data were collected from the electronic patient records as well as the paper charts. The extracted variables included demographics (age, sex), past medical history (previous history of admissions/antibiotic-resistant organisms, comorbidities), infectious disease diagnosis and treatment characteristics (antimicrobial name, class, and duration), documented follow-up plan at discharge, and outcome (readmission/return to emergency department, antimicrobial complications). For patients who underwent multiple courses of OPAT within the study period, the first hospitalization associated with OPAT was used. Antibiotic-resistant organisms were defined as methicillin-resistant* Staphylococcus aureus* (MRSA), vancomycin-resistant* Enterococcus* (VRE), extended-spectrum *β*-lactamase* Escherichia coli* and* Klebsiella* (ESBL), and carbapenemase-producing Enterobacteriaceae (CPE). Data collection was not limited to events experienced at SHSC, as electronic hospitalization and emergency department data were also available from five other major institutions in the Toronto area.

The primary outcome was all-cause readmission or emergency department visit within 60 days of discharge. Descriptive statistics were performed and mean and standard deviations were used to summarize normally distributed data, whereas the median and interquartile range were reported for non-normally distributed variables. Differences in patient, infection, and treatment characteristics among those with and without return visits were examined using chi-square tests for categorical variables and *t*-tests for normally distributed continuous variables.

## 3. Results

A total of 104 patients were found to be eligible for this study. Their demographics and clinical characteristics are listed in Table 1. Most of the patients were male (63%) and the median age was 63 years (interquartile range: 43 to 74 years). The median duration of hospital stay was seven days (interquartile range: 6 to 10 days), and 57% of the patients had a previous admission to hospital within the past 12 months. The most common comorbidities included hypertension (44%) and diabetes (28%). Twenty-seven patients had a history of cancer (26%) and five of these patients had been receiving chemotherapy or radiotherapy around the time of OPAT. Almost a quarter of the patients (23%) also had a history of infection or colonization with antibiotic-resistant organisms.

The most common infectious syndromes for which patients received OPAT included surgical site infections (33%), osteoarticular infections (28%, including hardware-related infections), and bacteremia (21%) (diagnoses not mutually exclusive) (Table 1). Of the 34 patients who had surgical site infections, 26 cases were classified as having deep or organ/space infections. While the majority of the patients were treated with just one drug for the duration of OPAT, some patients received up to four antimicrobials (oral and parenteral) concurrently. A total of 19 unique antimicrobials were administered in this study. The most frequently prescribed antimicrobials were ceftriaxone (21%) and cefazolin (20%), followed by piperacillin-tazobactam (14%) and cloxacillin (14%) (Table 1). A large proportion of patients received a peripherally inserted central catheter for vascular access (86%). Alternative routes such as Hickman and Port-A-Cath were used by one patient each, and the remaining 13 patients used their existing peripheral intravenous lines (13%) (data not shown).

According to documented discharge planning, 68% of the patients were intended to be followed by the admitting service, while 56% had follow-up arranged with an infectious diseases (ID) specialist (Table 1). Nearly all of the patients who were admitted under a surgical specialty were asked to follow up with the admitting service (93%). In contrast, patients under the medical service only required follow-up with the admitting team 38% of the time (data not shown).

**Figure 1:**
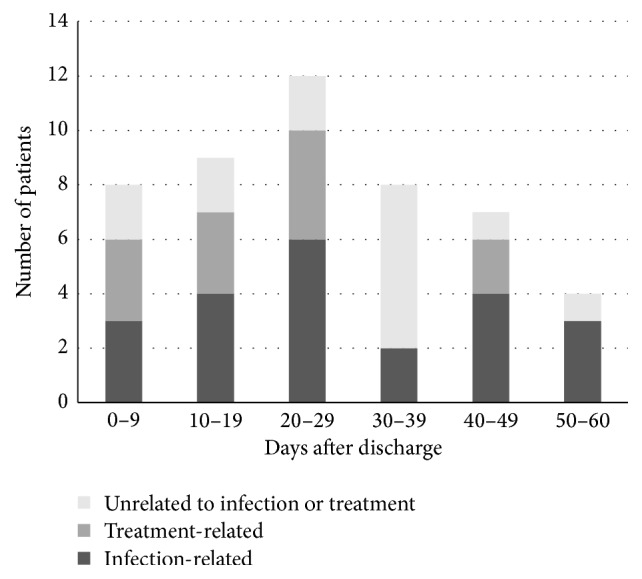
Time to readmission or emergency department use after discharge, grouped by cause. Infection-related causes include relapse or treatment failure. Treatment-related causes refer to complications related to OPAT, such as catheter problems or allergic/adverse drug reactions. Overall median time to event was 25 days (interquartile range: 13 to 38 days).

In the 60 days following discharge, 43% of the patients visited the emergency department, while 26% were readmitted. Almost half of these patients returned because of infection relapse (35%) or treatment failure (13%). A number of patients also returned due to treatment complications (24%), such as allergic reactions or disrupted venous access. Therefore, a large proportion of return visits (71%) were related to either the underlying infection or complications of the OPAT treatment itself; only a minority of return visits (29%) were unrelated (Table 1). The median time to return was 25 days (interquartile range: 13 to 38 days) and this did not differ appreciably by cause (Figure 1).

Patients who returned to the hospital were more likely to have a history of diabetes (38 versus 20%, *p* = 0.04) or cancer (35 versus 18%, *p* = 0.04). A diagnosis of urinary tract infection (25 versus 7%, *p* = 0.01) was also disproportionately more common among patients with return visits to the hospital. Documented plans for ID clinic follow-up were similar among patients with and without readmission (56% versus 55%, *p* = 0.93) (Table 1).

## 4. Discussion

In this study, the clinical characteristics and outcomes of patients who received OPAT at a single tertiary centre within a one-year period were analyzed. Approximately two patients per week were discharged with OPAT at SHSC, while only approximately half of these patients were referred to the ID service for follow-up. Nearly 50% of the patients were readmitted or returned to the emergency department shortly after discharge, usually for concerns related to their underlying infection or its treatment.

Similar to the 2013 study by Allison et al., the OPAT population at SHSC had a slight male predominance and patients were commonly treated for bacteremia or bone and soft tissue infections [11]. Antimicrobial selection was also comparable to the literature, with cephalosporins being the drug of choice in many cases [11, 12]. The readmission rates for OPAT reported in the literature range from 6% for skin and soft tissue infections to 50% for prosthetic joint infections [8, 9]. In our study, the 60-day all-cause readmission rate in a general sample of OPAT patient was 26%. This is comparable to the outcomes of two recent studies, which reported 30-day readmission rates of 26% and 20.5%, respectively [11, 12]. These studies did not examine emergency department visits, but we chose to include this endpoint because it is another important indicator of undesirable patient outcomes and potentially preventable health care expenses. Of note, 71% of all events (i.e., readmission/emergency department use) could be attributed to issues directly related to OPAT. Some of these events may be preventable through diligent follow-up and this highlights an opportunity for quality improvement. Although planned follow-up with an ID physician was not associated with a reduced risk of readmission/ER visit in this study, this may reflect selection bias if more complicated patients were referred for specialty care. Our sample size precluded the opportunity for multivariate analysis to examine this hypothesis. Furthermore, many of the patients who did not have ID follow-up were actually seen by the inpatient consult team prior to discharge (overall, approximately 75% of the patients had an ID consult as an inpatient).

In addition to sample size, other limitations of this study include the retrospective study design. We collected most of the data from discharge summaries and, when available, follow-up clinic notes; however, the plan at discharge may not always reflect the actual course of care. For instance, patients may not have been compliant with follow-up or may not have completed their scheduled course of antimicrobials. Our study may have also been influenced by incomplete follow-up data. Although the electronic system allowed us to capture readmission/emergency department events from six major hospitals in the region, some events may have been missed if patients visited a peripheral institution instead.

In conclusion, OPAT at SHSC is currently arranged at the discretion of each physician, and a significant proportion of these patients experience unplanned health care encounters soon after discharge. The creation of a formal OPAT clinic to coordinate structured monitoring may streamline the care of these patients and reduce unwanted outcomes. The results of this study have contributed to the funding and development of a one-year pilot OPAT program at SHSC, and a prospective study is planned to analyze its effectiveness.

## Figures and Tables

**Table 1:** OPAT patient characteristics and outcomes.

Variables	Total (*n* = 104)	Return to ED or readmission (*n* = 48)	No return to ED or readmission (*n* = 56)	*p* value
Age, years [median (IQR)]	63 (43–74)	64 (43–74)	61 (45–75)	0.95
Male	65 (63%)	28 (58%)	37 (66%)	0.42
Length of stay, days [median (IQR)]	7 (6–10)	7 (6–10)	7 (6–10)	0.28
Admitting service				
Medical	47 (45%)	23 (48%)	24 (43%)	0.61
Surgical	57 (55%)	25 (52%)	32 (57%)	0.61
Prior admission within past 12 months	59 (57%)	31 (65%)	28 (50%)	0.13
History of antibiotic-resistant organisms	24 (23%)	13 (27%)	11 (20%)	0.37
Comorbidities				
Hypertension	46 (44%)	24 (50%)	22 (39%)	0.27
Diabetes	29 (28%)	18 (38%)	11 (20%)	0.04
Gastrointestinal disease	29 (28%)	16 (33%)	13 (23%)	0.25
Cancer	27 (26%)	17 (35%)	10 (18%)	0.04
Coronary artery disease	23 (22%)	7 (15%)	16 (29%)	0.09
Peripheral vascular disease	13 (13%)	6 (13%)	7 (13%)	1.00
Arrhythmia	12 (12%)	5 (10%)	7 (13%)	0.74
Documented follow-up plan at discharge				
Admitting service	71 (68%)	32 (67%)	39 (70%)	0.75
Infectious diseases	58 (56%)	27 (56%)	31 (55%)	0.93
Family physician	35 (34%)	13 (27%)	22 (39%)	0.19
Most common antimicrobials^1^				
Ceftriaxone	22 (21%)	10 (21%)	12 (21%)	0.94
Cefazolin	21 (20%)	8 (17%)	13 (23%)	0.41
Piperacillin-tazobactam	15 (14%)	4 (8%)	11 (20%)	0.10
Cloxacillin	15 (14%)	10 (21%)	5 (9%)	0.08
Vancomycin	14 (13%)	6 (13%)	8 (14%)	0.79
Ertapenem	13 (13%)	8 (17%)	5 (9%)	0.23
Syndrome				
Surgical site infection	34 (33%)	14 (29%)	20 (36%)	0.48
Osteoarticular infection^2^	29 (28%)	13 (27%)	16 (29%)	0.87
Bacteremia	22 (21%)	14 (29%)	8 (11%)	0.06
Cellulitis	18 (17%)	9 (19%)	9 (16%)	0.72
Urinary tract infection	16 (15%)	12 (25%)	4 (7%)	0.01
Endocarditis	7 (7%)	4 (8%)	3 (5%)	0.70
Patient outcomes^3^				
No events	56 (54%)	—	56 (100%)	—
Events due to infection relapse	17 (16%)	17 (35%)	—	—
Events due to treatment failure	6 (6%)	6 (13%)	—	—
Events due to OPAT-related complications	11 (11%)	11 (23%)	—	—
Events due to unrelated causes	14 (13%)	14 (29%)	—	—

Values are expressed as number (%) unless otherwise stated.

^1^Less commonly used antimicrobials included ciprofloxacin, metronidazole, meropenem, clindamycin, fluconazole, cephalexin, rifampin, ceftazidime, ampicillin, penicillin G, nitrofurantoin, doxycycline, and voriconazole.

^2^Including 4 cases of diabetic foot infections and 1 case of prosthetic joint infection.

^3^Event defined as readmission or emergency department (ED) visit within 60 days of discharge.

IQR: interquartile range.
